# Serum IP-10 and IL-17 from Kawasaki disease patients induce calcification-related genes and proteins in human coronary artery smooth muscle cells in vitro

**DOI:** 10.1186/s13578-020-00400-8

**Published:** 2020-03-11

**Authors:** Shun-Fu Chang, Shih-Feng Liu, Cheng-Nan Chen, Ho-Chang Kuo

**Affiliations:** 1 Department of Medical Research and Development, Chiayi Chang Gung Memorial Hospital, Chiayi, Taiwan; 2 Department of Respiratory Therapy, Kaohsiung Chang Gung Memorial Hospital, Kaohsiung, Taiwan; 3 Division of Pulmonary & Critical Care Medicine, Department of Internal Medicine, Kaohsiung Chang Gung Memorial Hospital and Chang Gung University College of Medicine, Kaohsiung, Taiwan; 4 Department of Biochemical Science and Technology, National Chiayi University, Chiayi, 600 Taiwan; 5 Department of Pediatrics and Kawasaki Disease Center, Kaohsiung Chang Gung Memorial Hospital, #123, Da-Pei Rd., Niaosong, Kaohsiung, 83301 Taiwan; 6 Chang Gung University College of Medicine, Taoyuan, Taiwan

**Keywords:** Bone morphogenetic protein 6, Calcification, Interferon-γ-inducible protein 10, Interleukin 17, Smooth muscle cells

## Abstract

**Background:**

Kawasaki disease (KD) is one of the major causes of heart disease and vasculitis in children under 5 years old in the world. Clinical evidence has shown that coronary artery calcification may develop in KD patients, however the mechanism has not been elucidated. Previous studies have found that interferon-γ-inducible protein (IP)-10 and interleukin (IL)-17 can be elevated and may play a role in KD development and coronary artery lesion formation. The purpose of this in vitro study was to investigate the possible role of plasma circulating IP-10 and IL-17 of KD patients in vascular calcification development and its underlying mechanism.

**Result:**

Human coronary artery smooth muscle cells (HCASMCs) were used in this study. We found that HCASMCs treated with IP-10/IL-17-containing KD serum and co-treated with IP-10/IL-17 recombinant proteins could induce a phenotype that may promote vascular calcification by the bone morphogenetic protein (BMP) 6 autocrine effect. Moreover, the BMP6 autocrine stimulation in IP-10/IL-17 co-treated HCASMCs could upregulate the smad1/5-runx2 signaling activation, thus increasing the expression of bone matrix-related proteins, i.e., osteopontin, osteocalcin, and alkaline phosphatase.

**Conclusions:**

The presented in vitro results provided new insights into the comprehension of the pathogenesis of vascular calcification in SMCs in KD progression. Although additional in vivo experimental models should be completed to confirm the in vivo relevance of these in vitro findings, the results related to the autocrine role of BMP6 may provide a new direction for theranostic drug development to treat KD.

## Background

Kawasaki disease (KD) has been indicated as one of the causes of heart disease and vasculitis in infants and children under 5 years of age in the world. Regarding the principal clinical symptoms, KD can result in lesion development in the arterial vessels, particularly the coronary arteries, if KD patients are not treated in a timely manner. Such vessel lesions may include aneurysms, stenosis, thrombosis, and/or myocardial infarction [1–6]. While the symptoms and pathogenesis of KD have received more and more attention, the precise mechanisms are still debated. This uncertainty is due to the incidence of KD potentially being affected by seasonal, regional, hereditary, and age variations. A more reasonable and acceptable speculation regarding the development of KD was recently suggested, wherein KD may be initiated in patients with a genetic predisposition to infectious agents [1–6]. Furthermore, growing clinical and laboratory evidence has also indicated that the plasma levels of many cytokines and immunoregulatory molecules, including interleukin (IL)-6, -17, -18, and interferon-γ-inducible protein (IP) 10, are related to KD development and coronary artery lesion formation [7–13]. Therefore, these newly discovered molecules have been further investigated to determine their potential role as targets in the early diagnosis and/or treatment of KD patients.

Vascular calcification is a mineralization phenomenon of the vessel walls. In this event, vascular smooth muscle cells (VSMCs) express, secrete, and deposit bone matrix-related proteins in the vessel wall. Therefore, vascular calcification has also been indicated as a process of the phenotypic switching of VSMCs into osteoblast-like cells [14–16]. Bone morphogenetic proteins (BMPs) have been well-demonstrated to be the major inducer during bone formation. In the past two decades, BMPs (BMP2, 4, and/or 6) have also been found to have important roles in the development of various vascular diseases, including atherosclerosis and vascular calcification [14, 15, 17–25]. The most direct evidence is the presence of BMPs in vessel lesions and atherosclerotic plaques. Moreover, accumulating evidence has also indicated the inducer role of BMPs in regulating the expression of bone matrix-related proteins in the VSMCs of vessel walls [21, 26]. For KD development in patients, coronary arteritis can be initiated within 6 to 8 days, and then inflammation can subsequently be induced [1–6]. During this process, the abnormal activation and accumulation of monocytes/macrophages occur, thus changing the physiological activities of endothelial cells (ECs) and SMCs. These pathogenic phenomena have been indicated to resemble those of atherosclerosis development. Moreover, accumulating clinical data has also found that KD patients with large aneurysms may be prone to the formation of coronary artery calcification [27–29]. However, the mechanism of vascular calcification within KD progression is still unclear.

Our prior studies have shown that serum IP-10 and IL-17 levels are usually elevated in acute KD and may play a role in the disease pathogenesis [12, 13]. In this study, we investigated whether the serum IP-10 and IL-17 of KD patients could regulate the development of vascular calcification, as well as the underlying mechanism. We found that the serum IP-10 and IL-17 of KD patients could significantly initiate a calcification phenomenon in human coronary artery SMCs (HCASMCs) by BMP6 autocrine stimulation. It was shown that BMP6 could further activate smad1/5 signaling and transcription factor-runx2 to upregulate the osteogenic gene/protein expression in HCASMCs. Our in vitro findings indicated that vascular calcification could be a complication of coronary arteritis in KD patients. Furthermore, the mechanism clarifying the BMP6 autocrine effect in HCASMCs could serve as a novel and potential investigative target for the theranostic drug development of KD in the future.

## Results

### IP-10/IL-17 secreted in KD plasma prompts the calcification of HCASMCs

**Fig. 1:**
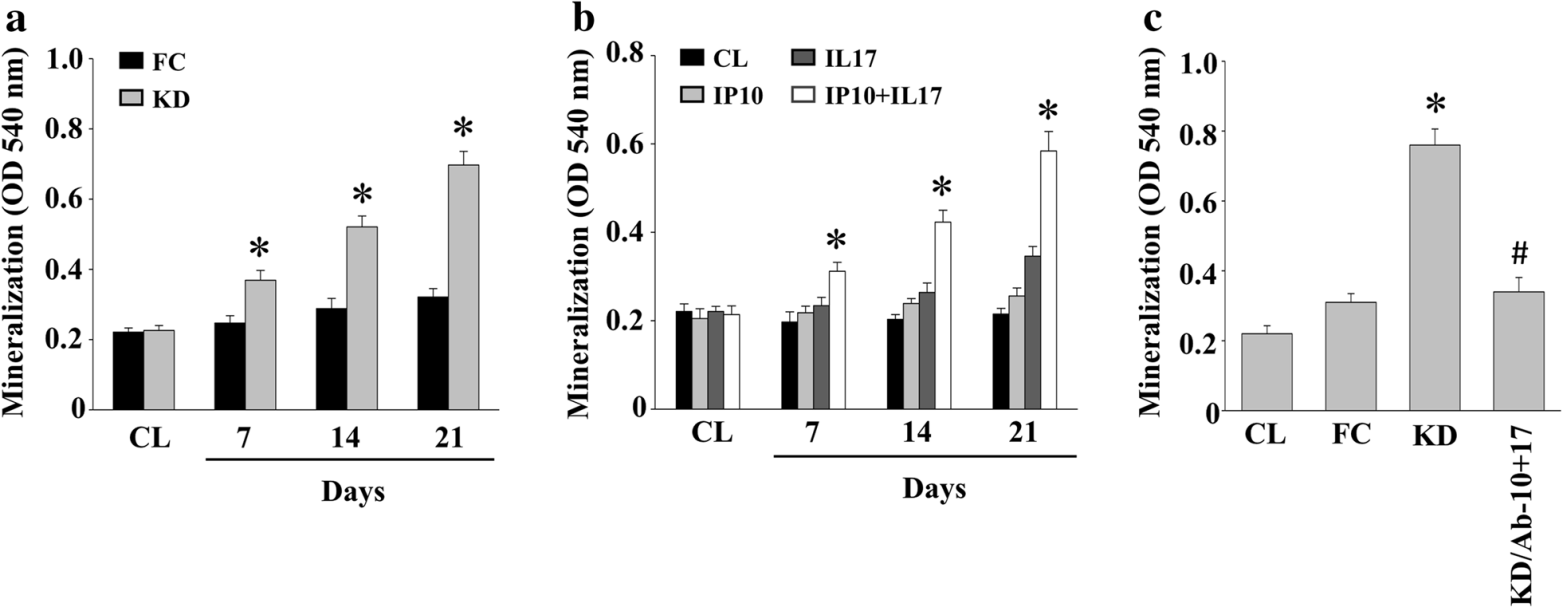
IP-10/IL-17 secreted in the KD plasma initiates the calcification of HCASMCs. **a** HCASMCs were either used as controls or were treated with either FC or KD plasma for 7, 14, or 21 days; **b** the HCASMCs were either maintained as controls or were treated with IP-10, IL-17, or both, for 7, 14, or 21 days; **c** the HCASMCs were either maintained as controls or were treated with FC or KD plasma, or KD plasma co-mixed with IP-10 and IL-17 blocking antibodies, for 21 days; **a**–**c** the calcification of the HCASMCs was analyzed using ARS stain. All data were mean ± SEM from three independent experiments; * *P* < 0.05 vs. control cells

To determine the pathogenesis of vascular SMCs in KD, HCASMCs were maintained as controls or were treated with either febrile children (FC) (IP-10 and IL-17 levels of 552 ± 72.6 pg/mL and 6.62 ± 1.56 pg/mL, respectively) or KD (IP-10 and IL-17 levels of 3126 ± 206.4 pg/mL and 26.3 ± 4.2 pg/mL, respectively) plasma for seven, 14, or 21 days. We then analyzed the calcification of HCASMCs through an ARS stain. The cells treated with KD plasma showed a significant increase in HCASMC calcification in a time-dependent manner compared to the control cells (Fig. 1a). The cells treated with FC plasma demonstrated no effect. We further determined whether IP-10 and/or IL-17 of KD influenced the development of HCASMC calcification. The cells were either maintained as controls or were treated with IP-10, IL-17, or both, for seven, 14, or 21 days, and were then analyzed for the calcification of HCASMCs through an ARS stain. We observed that HCASMC calcification was only initiated when the cells were co-treated with IP-10 and IL-17 compared to the control and the IP-10 only- or IL-17 only-treated cells (Fig. 1b). Furthermore, the KD plasma mixed with IP-10- and IL-17-blocking antibodies was also capable of reducing the HCASMC calcification levels of KD plasma induction (Fig. 1c).

### KD plasma induced osteopontin (OPN), osteocalcin (OCN), and alkaline phosphatase (ALP) expressions in HCASMCs

**Fig. 2:**
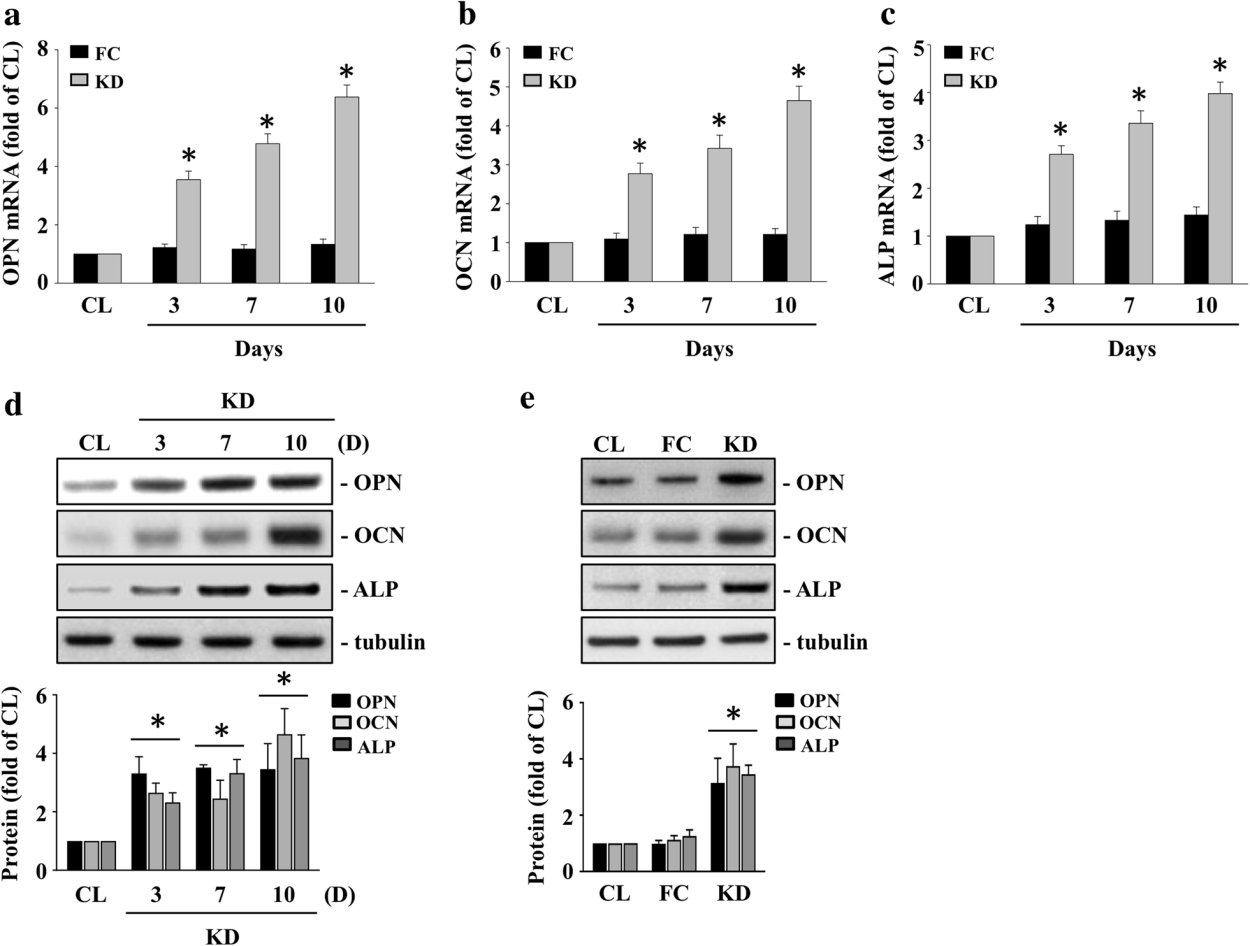
KD plasma induces OPN, OCN, and ALP expressions in HCASMCs. The HCASMCs were either maintained as controls or were treated with either FC or KC plasma for 3, 7, or 10 days, after which we examined the mRNA expressions of OPN (**a**), OCN (**b**), and ALP (**c**) using real-time PCR and the protein expressions of OPN, OCN, and ALP (**d**–**e**) using the western blot test. The data in (**a**–**e**) are mean ± SEM from three independent experiments. * *P* < 0.05 vs. control cells. The results in (**d**–**e**) are representative of three independent experiments with similar results

The expressions of bone matrix-related proteins, i.e., OPN, OCN, and ALP, in VSMCs were considered as the development of vascular calcification [16]. Therefore, we examined whether KD plasma could induce bone matrix-related proteins, i.e., OPN, OCN, and ALP, expressions in HCASMCs. The HCASMCs were either maintained as controls or were treated with either FC or KC plasma for 3, 7, or 10 days. The cells treated with KD plasma significantly induced OPN, OCN, ALP mRNA (Fig. 2a–c), and protein (Fig. 2d–e) expressions within 3 days and persisted for 10 days in the HCASMCs when compared to the control and the FC plasma-treated cells.

### IP-10/IL-17 co-treatment induces OPN, OCN, and ALP expressions in HCASMCs

**Fig. 3:**
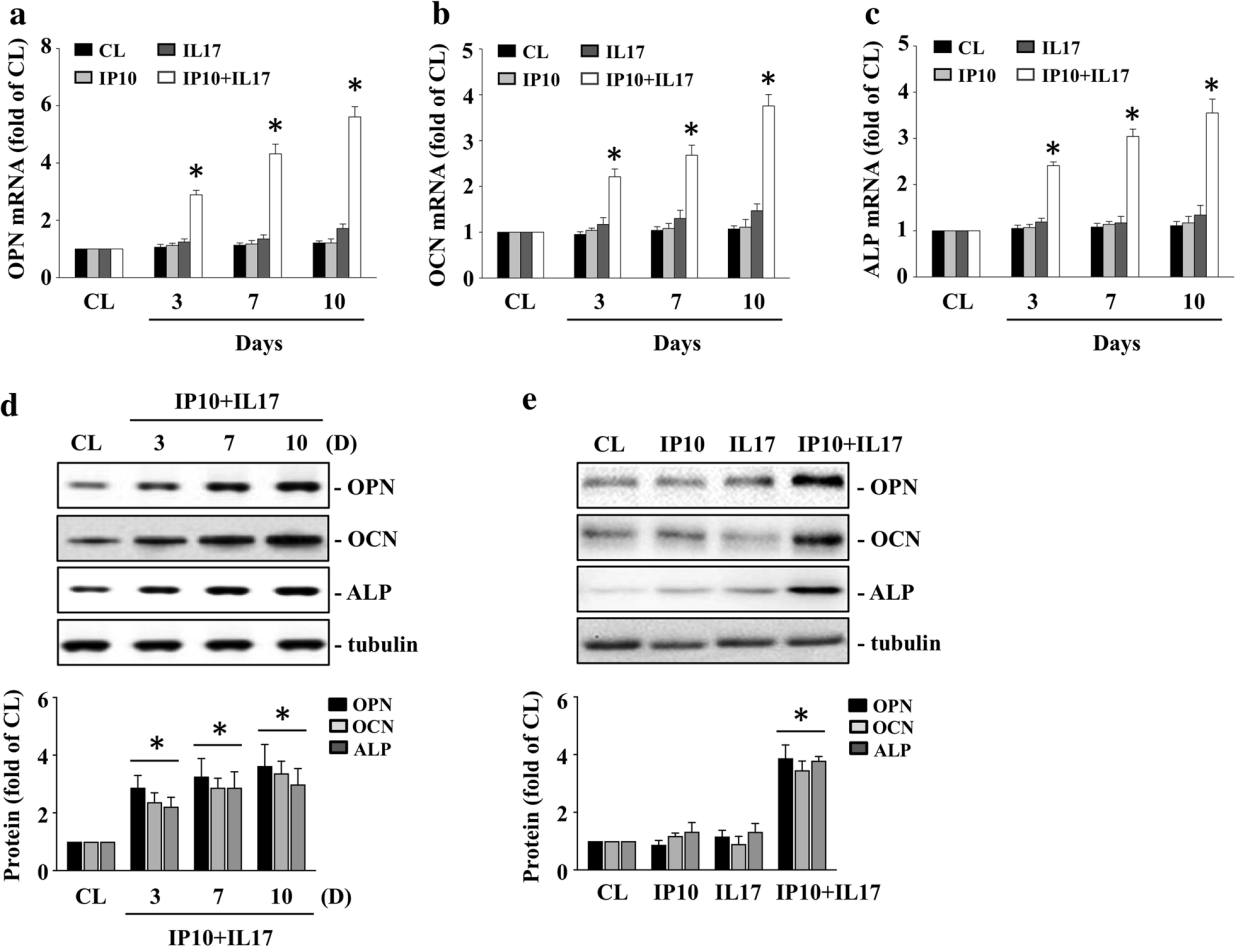
IP-10/IL-17 co-treatment induces OPN, OCN and ALP expressions in HCASMCs. The HCASMCs were maintained either as controls or were treated with IP-10, IL-17, or both, for 3, 7, or 10 days, after which we examined the mRNA expressions of OPN (**a**), OCN (**b**), and ALP (**c**) using real-time PCR and the protein expressions of OPN, OCN, and ALP (**d**–**e**) using the western blot test. The data in (**a**–**e**) are mean ± SEM from three independent experiments. * *P* < 0.05 vs. control cells. The results in (**d**–**e**) are representative of three independent experiments with similar results

We further examined whether IP-10 and IL-17 co-treatment was also capable of inducing OPN, OCN, and ALP expressions in HCASMCs. The HCASMCs were either maintained as controls or were treated with IP-10, IL-17, or both, for 3, 7, or 10 days. The cells co-treated with IP-10 and IL-17 significantly induced OPN, OCN, ALP mRNA (Fig. 3a–c), and protein (Fig. 3d–e) expressions within 3 days and persisted for 10 days in the HCASMCs when compared to the control and the IP-10 only- or IL-17 only-treated cells.

### IP-10/IL-17 co-treatment-initiated HCASMC calcification is mediated by the BMP6 autocrine effect

**Fig. 4:**
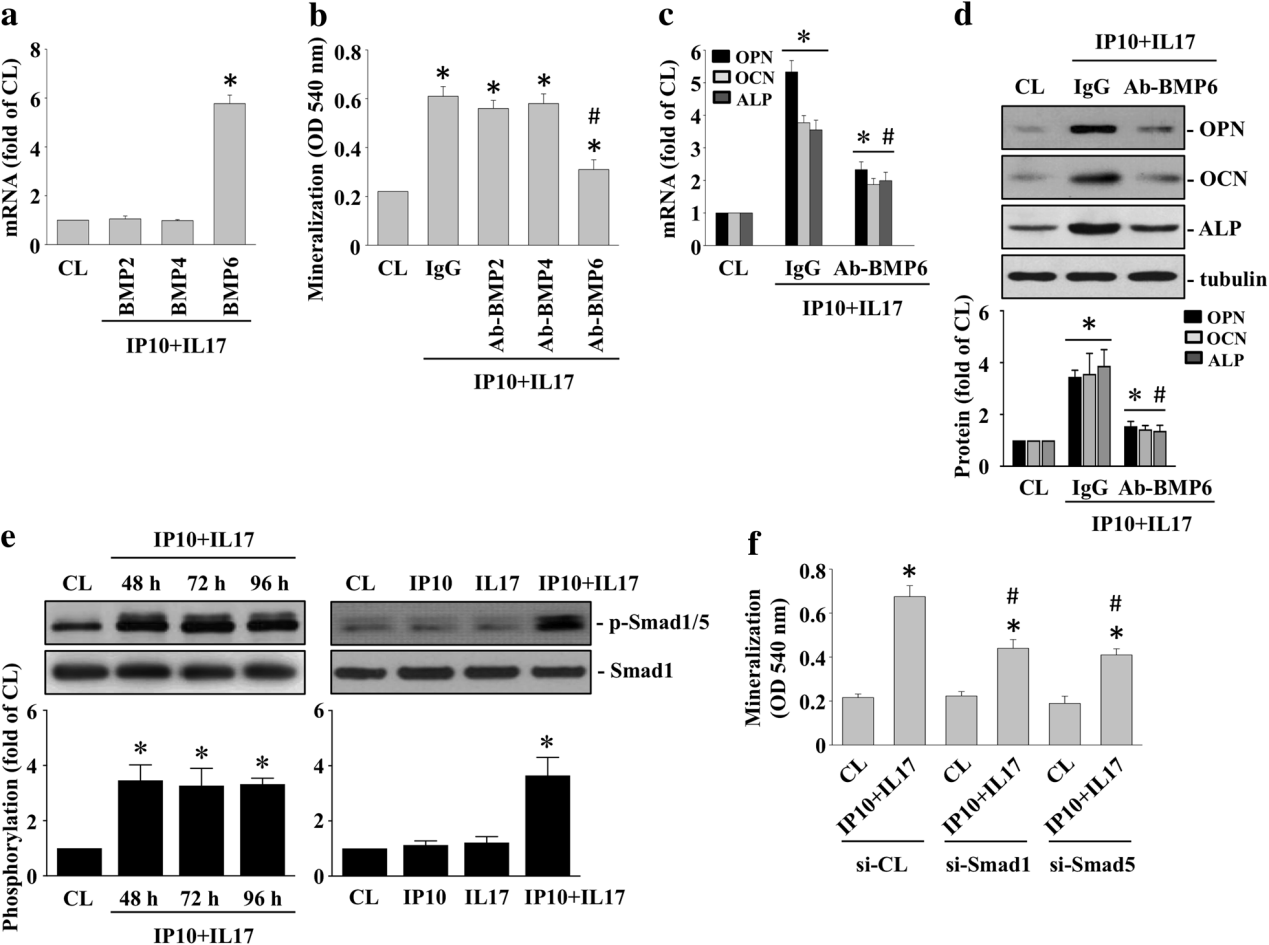
IP-10/IL-17 co-treatment-initiated HCASMC calcification is mediated by the BMP6 autocrine effect. **a** The HCASMCs were either maintained as the control or were co-treated with IP-10 and IL-17 for 3 days, after which we examined the BMP2/4/6 mRNA expression in the HCASMCs; **b**–**d** the HCASMCs were either maintained as the control or were pre-treated with IgG, BMP2-, BMP4-, or BMP6-blocking antibodies and then co-treated with IP-10 and IL-17; **b** HCASMC calcification was analyzed using ARS stain; **c** we examined the mRNA expressions OPN, OCN, and ALP using real-time PCR; **d** the protein expressions of OPN, OCN, and ALP were examined using the western blot test; **e** the HCASMCs were either maintained as the control or were treated with IP-10, IL-17, or both, for 48, 72, or 96 h, after which we examined the smad1/5 phosphorylation using the western blot test; **f** the HCASMCs were pretreated with control-, smad1- or smad5-specific siRNA and then either maintained as the control or co-treated with IP-10 and IL-17 for 14 days. The calcification of the HCASMCs was analyzed using ARS stain. The data in (**a**–**f**) are mean ± SEM from three independent experiments. * *P* < 0.05 vs. control cells; # *P* < 0.05 vs. IgG/or si-CL/IP-10 and IL-17-co-treated cells. The results in (**d**–**e**) are representative of three independent experiments with similar results

BMPs are potential regulators of vascular calcification development. Therefore, we set out to determine whether IP-10/IL-17 co-treatment-initiated HCASMC calcification would occur by upregulating the BMPs in the HCASMCs. Cells were either maintained as the control or were co-treated with IP-10 and IL-17 for 3 days, after which we examined the BMPs’ mRNA expression in the HCASMCs. The cells co-treated with IP-10 and IL-17 significantly increased the mRNA expression of BMP6 in the HCASMCs compared to the control cells (Fig. 4a). Furthermore, the cells pretreated with BMP6-blocking antibodies significantly reduced the up-regulatory effects of IP-10 and IL-17 co-treatment on HCASMC calcification levels (Fig. 4b), as well as the mRNA (Fig. 4c) and protein (Fig. 4d) expressions of OPN, OCN, and ALP. Smad1/5 is BMPs-specific downstream signaling. We investigated whether smad1/5 could regulate IP-10/IL-17 co-treatment-initiated HCASMC calcification. The HCASMCs were maintained either as controls or were co-treated with both IP-10 and IL-17 for 48, 72, or 96 h. We then examined the smad1/5 phosphorylation. IP-10 and IL-17 co-treatment of the HCASMCs significantly increased smad1/5 phosphorylation within 48 h and persisted for 96 h compared to the control cells (Fig. 4e). Treatment of the HCASMCs with IP-10 alone or IL-17 alone did not increase smad1/5 phosphorylation (right panel in Fig. 4e). Furthermore, the HCASMCs pretreated with smad1- or smad5-specific siRNA and then co-treated with IP-10 and IL-17 for 14 days significantly inhibited IP-10/IL-17 co-treatment-initiated HCASMC calcification (Fig. 4f).

### Runx2 regulates the BMP6 autocrine effect on IP-10/IL-17 co-treatment-initiated OPN/OCN/ALP expression and HCASMC calcification

**Fig. 5:**
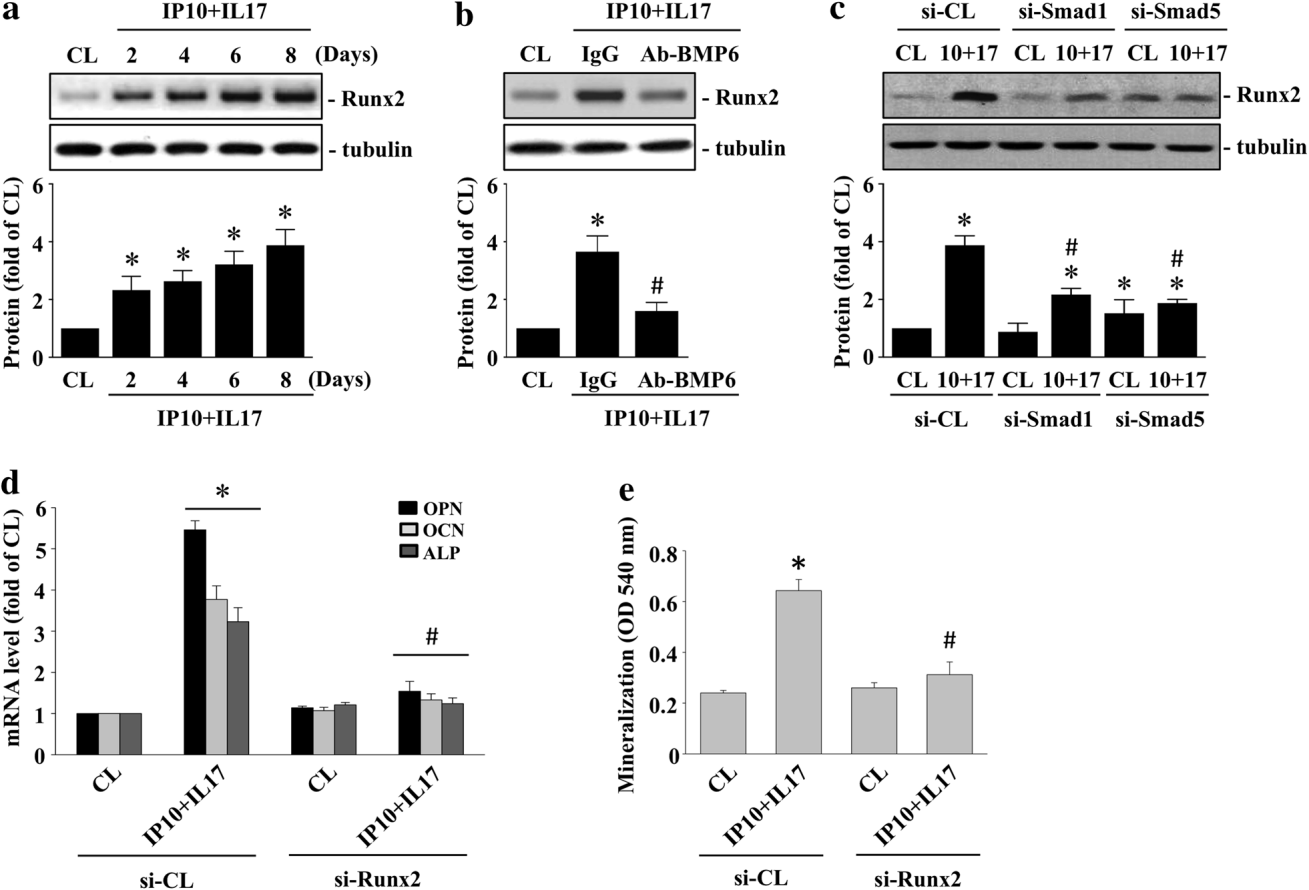
Runx2 regulates the BMP6 autocrine effect on the IP-10/IL-17 co-treatment-initiated OPN/OCN/ALP expression and calcification of HCASMCs. **a** The HCASMCs were either maintained as the control or were co-treated with IP-10 and IL-17 for 2, 4, 6, and 8 days; **b** the HCASMCs were either maintained as the control or were pre-treated with IgG or BMP6-blocking antibodies and then co-treated with IP-10 and IL-17 for 8 days; **c** the HCASMCs were pretreated with smad1- and smad5-specific siRNA and then either maintained as the control or co-treated with IP-10 and IL-17 for 8 days; **a**–**c** the expression of runx2 was examined using the western blot test; **d**–**e** the HCASMCs were pretreated with control- or runx2-specific siRNA and then maintained as either the control or co-treated with IP-10 and IL-17; **d** we examined the mRNA expressions of OPN, OCN, and ALP through real-time PCR; **e** the calcification of the HCASMCs was analyzed using ARS stain. The results in (**a**–**e**) are representative of three independent experiments with similar results. The data in (**d**–**e**) are mean ± SEM from three independent experiments. * *P* < 0.05 vs. siCL/control cells; # *P* < 0.05 vs. siCL/IP-10 and IL-17-co-treated cells

Runx2 is a BMP downstream transcription factor. Therefore, we investigated whether runx2 could regulate the BMP6 autocrine effect on the IP-10/IL-17 co-treatment-stimulated HCASMCs. The HCASMCs were maintained either as controls or were co-treated with both IP-10 and IL-17 for 2, 4, 6, and 8 days, after which we examined the runx2 expression. IP-10/IL-17 co-treatment of the HCASMCs significantly induced runx2 expression within 2 days and persisted for 8 days compared to the control cells (Fig. 5a). Pretreating cells with BMP6-blocking antibodies (Fig. 5b) or smad1- or smad5-specific siRNA (Fig. 5c) significantly inhibited IP-10/IL-17 co-treatment-induced runx2 expression in the HCASMCs. Furthermore, the HCASMCs pretreated with runx2-specific siRNA and then co-treated with IP-10 and IL-17 for either 10 or 14 days significantly inhibited IP-10/IL-17 co-treatment-initiated OPN, OCN, ALP mRNA expression (Fig. 5d), and HCASMC calcification (Fig. 5e).

### KD plasma has higher levels of secreted BMP6

**Fig. 6:**
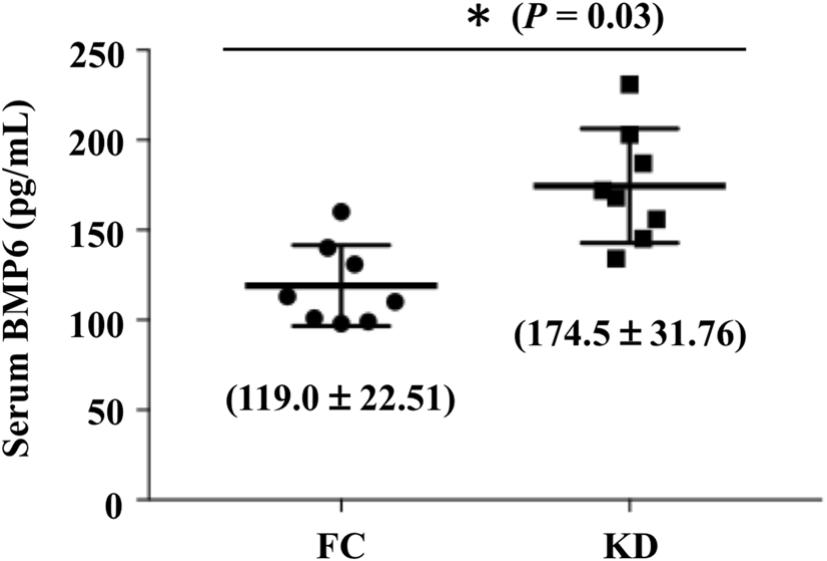
KD serum has higher levels of secreted BMP6. The plasma levels of BMP6 from eight non-KD febrile controls and eight KD patients were examined using an ELISA assay. * *P* < 0.03 vs. febrile controls

Finally, we investigated the plasma levels of BMP6 from eight non-KD febrile controls and eight KD patients using an ELISA assay to confirm the presented in vitro results. Our in vivo data showed that the average secretion levels of BMP6 in the plasma of the KD patients (174.5 ± 31.76 pg/mL) were higher than in the FC patients (119.0 ± 22.51 pg/mL) (Fig. 6).

## Discussion

**Fig. 7:**
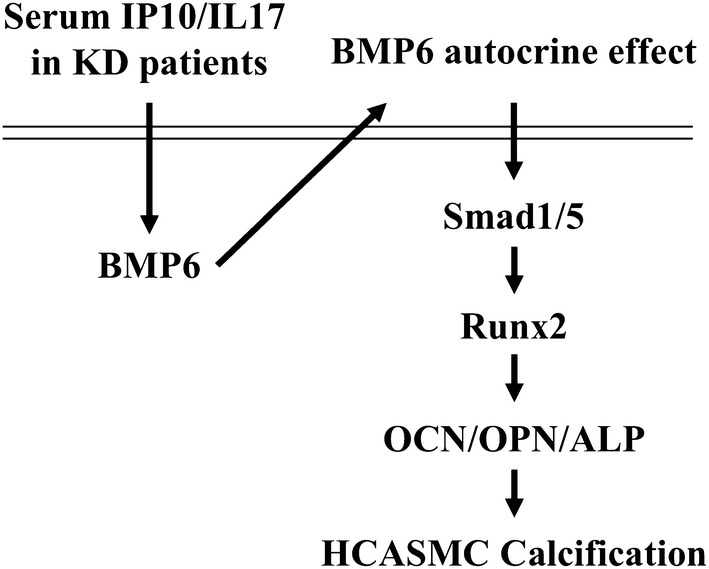
Schematic representation of the signaling pathways regulating KD plasma circulating IP-10 and IL-17 co-treatment-initiated vascular calcification in HCASMCs

This study found the effect of IP-10/IL-17-containing blood samples from KD patients on inducing genes/proteins associated with vascular calcification in HCASMCs and elucidated the underlying mechanism (Fig. 7). Our systematic evidence indicated that: (i) the circulating IP-10 and IL-17 in KD plasma had the capability to initiate the calcification of HCASMCs by inducing expressions of the osteogenic transcription factor, i.e., runx2, and subsequent bone matrix-related proteins, i.e., OPN, OCN, and ALP; (ii) the IP-10/IL-17 co-treatment-initiated the osteogenic switch development of HCASMCs, which was regulated by the BMP6 autocrine stimulation of the HCASMCs; (iii) HCASMC calcification of the KD serum induction required simultaneous stimulation of both IP-10 and IL-17. Treatment with IP-10 alone or IL-17 alone had no significant effect on HCASMC calcification. The presented in vitro findings provided new insights into the pathogenic effect of KD progression on vascular arteritis initiating vascular calcification in HCASMCs.

Coronary arteritis is the most common vascular complication of KD patients and coronary artery aneurysm is the most serious symptom, occurring in approximately 25% of KD patients who have not received treatment and in approximately 5% of KD patients who have been treated by intravenous immunoglobulin injections [30, 31]. In an aneurysm, the phenotype of ECs, myofibroblasts, and SMCs can be modulated by the inflammatory KD-secreted cytokines and vasoactive factors [1–5, 32, 33]. Previous studies have found that vasoactive factors, including vascular endothelial growth factors, nitric oxide, and platelet-derived growth factors, etc., may initiate the migration and proliferation of SMCs, as well as control the vascular permeability of KD progression [1–5, 32, 33]. Accumulating clinical data has further indicated that KD patients with large aneurysms may have an increased incidence of coronary artery calcification formation [27–29]. Moreover, it has been suggested that coronary artery calcification may be one of the sequelae of severe vasculitis occurring in acute KD patients [27–29]. The clinical symptoms of vascular calcification in KD patients has already been demonstrated, however its mechanism is still unclear. Although it still lacks an in vivo animal model or direct clinical evidence, the elucidation of the mechanism in this study could provide a new direction to explain the cause of KD vascular calcification.

VSMC calcification is one of the crucial components of cardiovascular disease [14, 16]. Previous studies have demonstrated that both IP-10 and IL-17 can be secreted into the plasma and subsequently may play a role in the development of coronary arteritis in KD [7–13]. Our present results further showed that the co-existence of IP-10 and IL-17 (but not treatment with just IP-10 or just IL-17) in KD plasma can initiate HCASMC calcification. Inflammation is an important factor in the formation of atherosclerosis, as well as KD. It has also been indicated that the coronary artery calcification of KD appears to be initiated by intensive inflammatory stimulation [29]. Both IP-10 and IL-17 can be secreted from immune cells, i.e., T helper cells, and are thus known to play an important role in autoimmune diseases [34–39]. Although still slightly controversial, recent studies have further indicated that IP-10 and IL-17 can be recognized as proinflammatory factors that may be involved in the development of coronary arterial disease, including aneurysms and atherosclerosis. [34–39]. Another study has also reported that both IL-17 and interferon-γ, an inducer of IP-10, can have a synergistic effect on VSMC pathogenesis, and that IL-17 may interact with interferon-γ to enhance the production of IP-10 [40]. Although we did not clarify the relationship between IP-10 and IL-17 in this study, including whether IP-10 is induced by interferon-γ, our present data support the possible synergistic regulatory role of IP-10 and IL-17 on pro-atherogenic stimulation in vascular cells. Moreover, the results indicated that the co-treatment of IP-10- and IL-17-specific blocking antibodies in KD patients’ plasma reduces the HCASMC calcification phenomenon induced by the KD patients’ plasma, which could potentially explain a new mechanism of how IP-10- and IL-17 might play a synergistic regulatory role in vascular calcification complications during KD progression.

Our have clarified that IP-10 and IL-17 co-treatment induces HCASMC calcification via the autocrine stimulation of BMP6 in HCASMCs. Furthermore, the BMP6-specific downstream smad1/5 and runx2 pathway can be activated to increase the expression of calcification-related proteins, i.e., OPN, OCN, and ALP, in IP-10 and IL-17 co-treated HCASMCs. BMPs are context-dependent growth factors and thus possess different functional activities in different cell types. In vascular tissues/cells, BMPs have also been demonstrated to have positive and negative functions for regulating vascular biology. In embryos, BMPs can control vascular angiogenesis and patterning [41, 42]. In contrast, BMP2/4/6, which does not involve BMP7 (as it represents atheroprotection), have been identified in atherosclerotic plaque and have been found to promote the dysfunction of ECs and SMCs [14, 15, 17–25]. Moreover, blocking BMP activity with the matrix Gla protein (MGP) in ApoE-/- transgenic mice may reduce the development of vascular calcification [43, 44]. In the present results, although higher levels of BMP6 were demonstrated in KD serum, we also found three samples from febrile patients that had similar results. It has been suggested that more serum samples from KD and febrile patients are needed to be counted and studied. Moreover, both KD and febrile disease have been considered as a kind of inflammation. Thus, our study also suggested that BMP6 may play a role in the inflammation regulation of KD (and even febrile disease), however this role needs to be further examined in the future. Overall, although the detailed mechanisms and roles of individual BMPs (BMP2, 4, and 6) in vascular calcification formation have not been completely identified, our results suggest another vascular calcification-promoting role of BMP6 in KD pathogenesis and might under the IP-10/IL-17 co-treatment condition.

## Conclusion

This in vitro study suggested that the serum IP-10 and IL-17 of KD patients could play a role in stimulating HCASMCs to induce a phenotype that promotes vascular calcification, which is regulated by the BMP6 autocrine stimulation and consequent smad1/5-runx2 signaling activation and bone matrix-related proteins, i.e., OPN, OCN, and ALP expressions. Additional in vivo experimental mouse models of KD and human studies will be needed to confirm the in vivo relevance of these in vitro findings. Therefore, this limitation will be addressed in our ongoing study and be further elucidated in future. Overall, this in vitro study could provide new insights into the pathogenesis of vascular calcification in VSMCs in KD progression.

## Methods

### Materials

IP-10, IL-17, and BMP6 ELISA kit, BMP2-specifc rabbit polyclonal antibody, BMP4-specific mouse monoclonal antibody, and BMP6-specific goat polyclonal antibody were come from the Biocompare (San Francisco, CA). Smad1/5-specific goat polyclonal antibody, ALP-specific rabbit polyclonal antibody, and OCN-/OPN-specific mouse monoclonal antibody was from the Santa Cruz Biotechnology (Santa Cruz, CA). pSmad1/5-/runx2-/tubulin-specific rabbit polyclonal antibody was from the Cell Signaling Technology (Beverly, MA). The control- and smad1-/smad5-/runx2-specific siRNAs were from the Thermo (Waltham, MA). All others reagents and chemicals were obtained from Sigma (St. Louis, MO).

### Patients

Blood samples from the children who met the KD criteria [45] were collected before intravenous immunoglobulin (IVIG) treatment at Kaohsiung Chang Gung Hospital. Blood samples from the children who did not met the KD criteria (acute fever for less than 5 days) and from an incomplete collection were excluded. The age-matched children who had febrile symptom and an upper and/or lower respiratory tract infection were indicated as the controls. Plasma was obtained from the patients’ blood sample within 1 day (centrifuge: 3000 rpm/10 min) and then stored in a − 80 °C until usage. The plasma IP-10 and IL-17 levels from all the patients’ blood samples were determined by ELISA assay. The average levels of IP-10 and IL-17 from KD patients are 3126 ± 206.4 pg/mL and 26.3 ± 4.2 pg/mL, respectively. The average levels of IP-10 and IL-17 from febrile patients are 552 ± 72.6 pg/mL and 6.62 ± 1.56 pg/mL, respectively. The Institutional Review Board (IRB) certificate (IRB No. 104-2726A3) for this study was reviewed and approved by the Chang Gung Memorial Hospital. We obtained the informed consent from patients’ parents or guardians before the study.

### Patient and public involvement

We did not involve patients or the public in our work.

### Cell culture

Human coronary artery SMCs (HCASMCs, ATCC^®^ PCS-100-021™) were purchased from the cell bank (ATCC, Rockville, MD). The cells were cultured in an FBS/antibiotics-containing F12K medium. In all the experiments of this study, only 5 passages of HCASMCs were used.

### SMC calcification assay

The mineralization level of HCASMCs were examined by ARS (Alizarin Red S) stain. ARS (an anthraquinone dye) is a calcium-deposition detective reagent. ARS stain has been widely used to analyze calcium-rich deposits in mineralizing tissues and cells for many decades [46–48]. After the treatment, the cells were fixed (formaldehyde) and then were stained with ARS reagent. These stained cells were further extracted by adding the acetic acid and then were neutralized by adding the ammonium hydroxide. Finally, the samples were centrifugated and the supernatants were collected and examined by using the colorimetric detection (405 nm).

### Real-time PCR

Real-time PCR experiments were carried out by using the SYBR Green kit (Thermo, Waltham, MA). The RNA samples were purified from the HCASMCs and were further converted into the cDNA by reverse-transcription PCR. The specific sequences of BMP2 primer (plus-CGCAGCTTCCACCATGAAGAA and minus-CCTGAAGCTCTGCTG AGGTGATA), BMP4 primer (plus-AGGAGCTTCCACCACGAAGAAC and minus-TGGA AGCCCCTTTCCCAATCAG), BMP-6 primer (plus-GTGAACCTGGTGGAGTACGACAA and minus-AGGTCAGAGTCTCTGTGCTGATG), ALP primer (plus-CTCCCAGTCTCATC TCCT and minus-AAGACCTCAACTCCCCTGAA), OPN primer (plus-GGACAGCCAG GACTCCATTG and minus-TGTGGGGACAACTGGAGTGAA), OCN primer (plus-GTG ACGAGTTGGCTGACC and minus-CAAGGGGAAGAGGAAAGAAGG), and GAPDH primer (plus-AGGTGAAGGTCGGAGTCAAC and minus-CCATGTAGTTGAGGTCAATG AAGG) were used in the real-time PCR. The expression level of GAPDH were use as the internal control.

### Western blot

Cell lysates were collected by lysing cells with 0.1% SDS-containing lysis buffer. The protein samples (25 µg/sample) were loaded and separated in 10% SDS-PAGE and then were transferred to a nitrocellulose paper (0.45 µm pore size). The nitrocellulose paper was further hybridized and analyzed with the indicated antibodies and the detection system.

### siRNA transfection

Cells were cultured in antibiotics-free medium over 16 h and then were transfected with the smad1-/smad5-/runx2-specific siRNA for 25 nM by using the RNAiMax Transfection kit. The knockdown efficiency of all of the investigated genes were demonstrated to have ~ 70% reduction.

### Elisa

The plasma BMP6 levels were examined by the ELISA kit. The clinical plasma samples from both the FC and KD patients were recruited and harvested. The plasma BMP6 levels were further examined by using the BMP6-specific antibodies and spectrophotometry.

### Statistical analysis

The results were shown as the mean ± SEM from at least three repeated experiments. The statistical analysis was completed using an independent student t-test for the two groups of data and analysis of variance (ANOVA) followed by Scheffe’s test for multiple comparisons. *P *< 0.05 was defined as being statistically significant. In all the assays, at least three individual experiments were carried out.

## Data Availability

The data that support the findings of this study are available from the corresponding author upon reasonable request.

## Abbreviations

KD: Kawasaki disease
IL: Interleukin
IP-10: Interferon-γ-inducible protein 10
HCASMCs: Human coronary artery smooth muscle cells
BMPs: Bone morphogenetic proteins
ECs: Endothelial cells
FC: Febrile children
ALP: Alkaline phosphatase
OPN: Osteopontin
OCN: Osteocalcin
IVIG: Intravenous immunoglobulin
ARS: Alizarin Red S
